# Early marriage and its associated factors among women in Ethiopia: Systematic reviews and meta-analysis

**DOI:** 10.1371/journal.pone.0292625

**Published:** 2023-11-22

**Authors:** Natnael Atnafu Gebeyehu, Molalign Melese Gesese, Kirubel Dagnaw Tegegne, Yenalem Solomon Kebede, Gizachew Ambaw Kassie, Misganaw Asmamaw Mengstie, Melkamu Aderajaw Zemene, Natnael Moges, Berihun Bantie, Sefineh Fenta Feleke, Tadesse Asmamaw Dejenie, Endeshaw Chekol Abebe, Denekew Tenaw Anley, Anteneh Mengist Dessie, Wubet Alebachew Bayih, Getachew Asmare Adella

**Affiliations:** 1 School of Midwifery, College of Medicine and Health Sciences, Wolaita Sodo University, Sodo, Ethiopia; 2 Department of Comprehensive Nursing, College of Medicine and Health Science, Wollo University, Dessie, Ethiopia; 3 Department of Medical Laboratory Science, College of Health Science, Debre or University, Debre Tabor, Ethiopia; 4 Department of Epidemiology and Biostatistics, School of Public Health, Wolaita Sodo University, Wolaita Sodo, Ethiopia; 5 Department of Biochemistry, College of Health Science, Debre Tabor University, Debre Tabor, Ethiopia; 6 Department of Public Health, College of Health Sciences, Debre Tabor University, Debre Tabor, Ethiopia; 7 Department of Pediatrics and Child Health Nursing, College of Health Sciences, Debre Tabor University, Debre Tabor, Ethiopia; 8 Department of Comprehensive Nursing, College of Health Sciences, Debre Tabor University, Debre Tabor, Ethiopia; 9 Department of Public Health, College of Health Sciences, Woldia University, Woldia, Ethiopia; 10 Department of Medical Biochemistry, College of Health Science, Gondar University, Gondar, Ethiopia; 11 Department of Maternal and Neonatal Nursing, College of Health Sciences, Debre Tabor University, Debre Tabor, Ethiopia; 12 School of Public Health, College of Health Science and Medicine, Wolaita Sodo University, Wolaita Sodo, Ethiopia; St. Paul’s Hospital Millennium Medical College, ETHIOPIA

## Abstract

**Background:**

Early marriage is defined as the union of one or both partners before reaching the age of 18 for the first time. This practice is widely prevalent in underdeveloped countries, particularly in Ethiopia, and has been observed to have detrimental effects on the educational and personal development of both male and female individuals.

**Methods:**

The present study conducted a comprehensive search of the Science Direct, Scopus, Google Scholar, EMBASE, and PubMed databases. The data were extracted using Microsoft Excel (version 14) and analyzed using STATA statistical software. To examine publication bias, a forest plot, rank test, and Egger’s regression test were utilized. Heterogeneity was assessed by calculating I^2^ and conducting an overall estimated analysis. Additionally, subgroup analysis was performed based on the study region and sample size. The pooled odds ratio was calculated.

**Results:**

Out of a total of 654 articles, 14 papers with 67,040 research participants were included in this analysis. The pooled prevalence of early marriage among women in Ethiopia was 56.34% (95% CI: 51.34–61.34), I^2^ = 78.3%). The Amhara region exhibited the highest prevalence of early marriage, with a rate of 59.01%, whereas the Oromia region demonstrated the lowest incidence, with a prevalence rate of 53.88%. The prevalence of early marriage was found to be 58.1% for a sample size exceeding 1000, and 50.9% for a sample size below 1000. No formal education (AOR = 5.49; 95%CI: 2.99, 10.07), primary education (AOR = 3.65; 95%CI: 2.11, 6.32), secondary education **(**AOR = 2.49; 95%CI: 1.60, 3.87), rural residency (AOR = 4.52; 95%CI: 1.90, 10.74) and decision made by parents (AOR = 2.44; 95%CI: 1.36, 4.39) were associated factors.

**Conclusion and recommendation:**

In Ethiopia, there was a high rate of early marriage among women. The research findings indicate that early marriage is more prevalent among mothers who possess lower levels of educational attainment, reside in rural areas, and are subject to parental decision-making. Our stance is firmly in favor of expanding the availability of maternal education and promoting urban residency. Furthermore, the promotion of autonomous decision-making by clients regarding their marital affairs is of paramount importance to family leaders.

## Introduction

The phenomenon of early marriage is characterized by the occurrence of marriage before the female partner’s physical, physiological, and psychological readiness for the responsibilities of marriage and childbirth, and before reaching the age of 18 years [1, 2]. This issue is of significant global concern, as it poses a threat to public health and constitutes a violation of human rights [3, 4]. In low- and middle-income countries, early marriage is particularly problematic, as it poses a threat to the health and rights of children [5]. Although the average age of marriage is increasing for both sexes, early marriage remains a problem in developing countries [6]. According to the United Nations Population Fund, in many parts of the developing world, nearly one in three girls marries in their teens [7]. However, rates vary widely between and within countries. The highest rates were reported in southern Asia (44%) and sub-Saharan Africa (39%) [8]. Six million girls in Ethiopia marry before the age of 15, and four out of ten young women marry before the age of 18 [9].

The negative consequences of child marriage can be considered at multiple levels for girls, their families, their offspring, and ultimately for the wider community in terms of social, health, and financial consequences [10, 11]. Hence, it can be posited that early marriage constitutes a significant public health concern that has deleterious effects on the growth and overall well-being of both mothers and children [12–21]. The heightened risk of school dropout among adolescent mothers further exacerbates the issue, as it precludes opportunities for personal development and educational advancement [22–24]. Moreover, young age mothers are at an increased likelihood of experiencing helplessness, social exclusion, poverty, limited access to familial and social networks, and a higher risk of divorce [25–27]. Furthermore, the offspring of adolescent mothers are more susceptible to newborn and childhood mortality and morbidity, nutritional deficiencies, low birth weight, and congenital anomalies [28–30].

Over the past few decades, a range of policies, methods, and initiatives have been examined at both international and regional levels to tackle the challenges associated with early marriage [31, 32]. In line with other nations, Ethiopia’s revised Family Law stipulates that individuals must be at least 18 years of age to enter into marriage [33]. Despite the existence of national regulations in Ethiopia, it is commonplace for girls under the age of 18 to be wed, with significant implications for many females [34].

The phenomenon of early marriage among women has been associated with a multitude of factors. These factors encompass the educational attainment of both women and their parents, the size of the family, the geographical location, the financial status of the household [35–39], the adherence to a particular religious faith [40], the awareness of the legal age of marriage, and the recognition of the potential consequences of early marriage [35].

To the best of our knowledge, what is needed is the current evidence that clarifies the impact of early marriage nationally. Although national-level research already exists, most of these studies have not produced consistent results on the magnitude and key predictors associated with early marriage. Consequently, applying these results to the problem of early marriage has been challenging. To address this issue, it was deemed necessary to consolidate the different results and compile the most reliable data on early marriage in Ethiopia. As a result, the findings of this study will provide program planners and policymakers with scientific evidence for protecting early marriage. In addition, the study’s findings will be able to address the problem of young women getting married too young and operational strategies, giving them the fundamental information they require for all young women.

## Methods

### Data synthesis and reporting

The present study involved the analysis of data derived from a solitary measurement outcome, specifically about early marriage. The findings were presented through the utilization of tables, textual descriptions, and a forest plot. Adhering to the standard PRISMA checklist guideline, a systematic review and meta-analysis were conducted to determine the pooled prevalence of early marriage among women in Ethiopia [41] (**S1 File**). The protocol for this systematic review and meta-analysis has been submitted to the International Prospective Register of Systematic Reviews (PROSPERO) with registration number CRD42023445490.

### Search strategy

We thought of employing PECO questions used for the explicit presentation of our review question and the clarification of the inclusion and exclusion criteria. These queries were made by combining the words and phrases below with the MeSH (Medical Subject Headings) and the Boolean operators "OR" and "AND".

### PECO guide

#### Population

All female individuals who have not yet reached the age of 18 were population.

#### Exposure

Every female who married before turning 18 years old was population underexposure.

#### Comparison

Girls under the age of 18 years who do not get married were used as a comparison group.

#### Outcome

Early marriage.

To find as many pertinent primary studies as feasible, we created the following review question using the modified PICO structure described above:

*Review question*. "What percentage of Ethiopian marriages occurs before age 18?

Articles on the prevalence of early marriage in Ethiopia were searched using international online databases (Pub Med, Science Direct, Scopus, EMBASE, and Google Scholar). The following keywords and search terms were used during the search: "Prevalence," early marriage," "child marriage," "short form of marriage," "short term of marriage," "determinant," "predictor," "factor," and "Ethiopia." Boolean operators like "OR" and "AND" were used to combine the search phrases as well as use them separately. The search was carried out from November 15, 2022, until December 21, 2022.

#### Study outcome

Young females who marry before turning 18 are said to have engaged in early marriage [3, 4]. If the women’s first marriage occurred before the age of 18, the outcome variable was dichotomized and coded as "yes," and if it occurred at or after the age of 18, it was "no."

### Inclusion and exclusion criteria

The papers that were included in this meta-analysis were those that were conducted in Ethiopia, were published in English, and had full texts that could be searched. Studies that included data on early marriage were also reported on. Qualitative studies, research from duplicated sources, and articles missing the complete text were all omitted from this systematic review and meta-analysis. The eligibility of the included articles in this study was determined using the COCOPOP (Condition, Context, and Population) paradigm. Married women made up the study population (POP), with the prevalence of early marriage serving as the condition (CO), and only studies carried out in Ethiopia serving as the context (CO).

#### Quality assessment

The present study employed a standardized quality appraisal checklist, which was developed by the Joanna Briggs Institute (JBI) [42], to assess the level of research. Two authors namely NAG and KDT conducted the appraisal independently. In the event of any disagreement that arose during the quality assessment, a dialogue was facilitated by the third author, GAA, to resolve the issue. Eight parameters on the critical analysis checklist have yes, no, uncertain, and not relevant boxes. The criteria include the following inquiries: (1). Where were the inclusion criteria for the sample clearly stated? (2). Were the study participants and, by extension, the environment thoroughly described? (3). Were the results of the exposure measurement valid and reliable? (4). Did the event meet the primary purpose and accepted standards? (5) Were confounding elements found? (6). Were confounding factor measures mentioned? (7). Were the outcomes actually and accurately measured? (8). Was the statistical analysis appropriate? Studies were considered low risk when they scored 50% and above on the quality assessment indicators (**S2 File).**

### Risk of bias assessment

The present study employed the bias assessment tool developed by Hoy et al. [43], which comprises ten items that evaluate four domains of bias, as well as internal and external validity. Two authors namely NAG and GAA independently assessed the included papers for potential bias. In the event of any disagreement arising during the risk of bias assessment, a discussion was led by the third author, KDT, to resolve the issue. Ultimately, a consensus was reached through mutual agreement. The first four items (1–4) of the tool were used to evaluate the presence of selection bias, non-response bias, and external validity, while the remaining six items (5–10) were used to assess the presence of measurement bias, analysis-related bias, and internal validity. Studies were categorized as having a "low risk of bias" if they answered "yes" to eight or more of the ten questions. Studies were classified as having a "moderate risk" if they answered "yes" to six to seven of the ten questions, while studies that answered "yes" to five or fewer of the ten questions were classified as having a "high risk" of bias (**S3 File**).

#### Data extraction

The process of extracting data in this study adhered to a standardized format developed by the Joanna Briggs Institute. Two authors namely NAG and KDT independently conducted the extraction of all pertinent data. In the event of any discrepancies that emerged during the extraction process, a discussion was facilitated by the third author, GAA, to arrive at a consensus. Given that this study did not employ a paper form, manual data extraction was utilized instead of an automated tool. The extracted data encompassed the first author’s name, year of publication, study region, study setting, study design, sample size, prevalence of early marriage, unadjusted odds ratio for variables, and paper quality.

#### Data analysis

The present study involved the extraction of relevant data from a Microsoft Excel spreadsheet, which was subsequently exported to STATA software version 14 for analysis. A meta-analysis was conducted using a weighted inverse variance random-effects model to generate a pooled odds ratio (OR). The presence of heterogeneity was assessed visually through a forest plot, which was utilized to estimate the pooled estimate of early marriage. Subgroup analysis was performed based on the study region and sample size. Sensitivity analysis was employed to evaluate the impact of individual studies on the overall prevalence estimate of the meta-analysis. The funnel plot was utilized to investigate potential publication bias, and Begg and Egger’s regression tests were employed to examine it more objectively. Cochran’s Q X^2^ test and I^2^ statistics were used to test for heterogeneity, estimate the total and residual heterogeneity, and measure the variability caused by heterogeneity [44]. Additionally, a univariate meta-regression analysis was conducted to explore the effects of sample size and publication year variations on between-study heterogeneity [45].

#### Patient and public involvement

The authors (NAG and KDT) collaborated with researchers from previous studies and public experts to develop the research questions and outcome measurements. As this study was a systematic review and meta-analysis based on publicly available data, patients and research participants were not involved in the design or analysis of the study. The results of this study will be communicated to patients and study participants through health education initiatives focused on early marriage. The dissemination of key findings will be facilitated through the distribution of brochures in the local language.

## Results

### Search results and study characteristics

**Fig 1:**
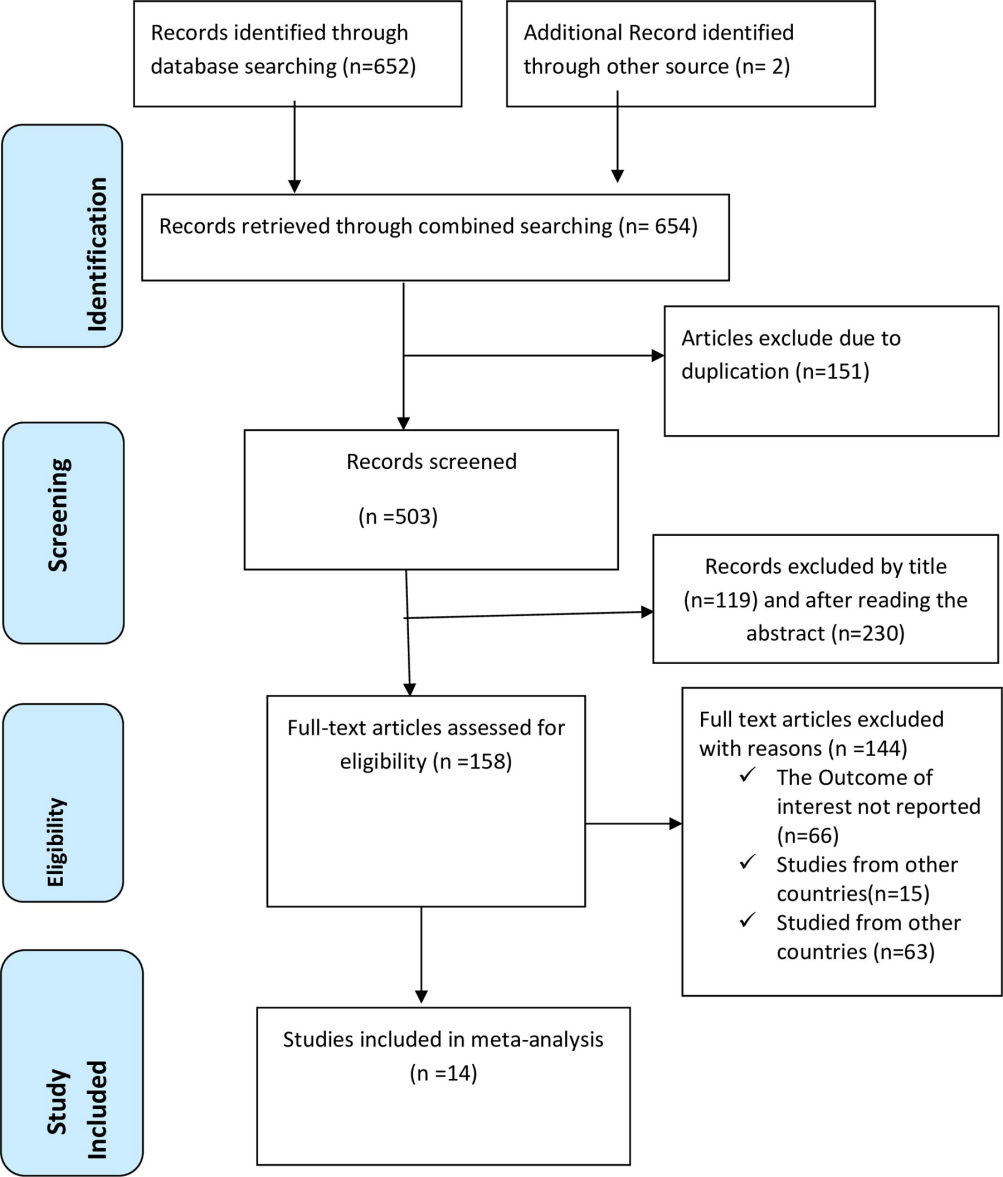
PRISMA flow chart displays the article selection process for the prevalence of early marriage among young women in Ethiopia.

A comprehensive search was conducted across multiple global web databases, namely PubMed, Scopus, EMBASE, Science Direct, and Google Scholar, resulting in the retrieval of 654 articles. Following the removal of duplicate research, 503 studies underwent a thorough screening process, which involved a complete evaluation of their titles and abstracts. Subsequently, 339 studies were excluded, and the remaining 158 studies were subjected to a full-text review. Upon completion of the review, 144 articles were excluded based on further criteria. The present systematic review and meta-analysis study included 14 papers [34, 46–58], which comprised a total of 67,040 study participants, by the pre-defined inclusion criteria (Fig **1**).

The present study employed a cross-sectional design in all of the studies that were included. Notably, all of the studies were community-based in nature. Specifically, the sample comprised 18 studies conducted in National [34, 49, 50, 54–58], three in Amhara [46, 47, 52], two in Oromia [48, 53], and one in Amhara, Southern Nations and Nationalities People’s Region, and Oromia region [51]. The sample sizes ranged from 373 to 11649, while the rate of early marriage ranged from 40% to 73%. To ensure the quality of the studies, the Joanna Briggs Institute (JBI) quality appraisal checklist was employed, and the findings indicated a low risk of bias **(Table 1).**

**Table 1:** Characteristics of studies included in the systematic review and meta-analysis of early marriage of women in Ethiopia.

Author/year	Region	Setting	design	Sample size	Prevalence	Mean age	Standard deviation	Quality
Muhammedawel Kasso/2017 48]	Oromia	Community	Cross-sectional	619	58.7%	30	30±9.2	Low-risk
Alem et.al/2020 [58]	National	Community	Cross-sectional	11649	NR	NR	NR	Low-risk
Tekile AK/2020 [47]	National	Community	Cross-sectional	1120	48.57%	15	15±8.4	Low-risk
Sileshi Workineh/2015 [46]	Amhara	Community	Cross-sectional	802	NR	NR	NR	Low-risk
Erulkar/2019 [34]	National	Community	Cross-sectional	2903	40%	NR	NR	Low-risk
Kassahun Tiruaynet/un-pub [49]	National	Community	Cross-sectional	2903	40.2%	15.02	15.02±1.65	Low-risk
kassie Wubet/un-pub [57]	National	Community	Cross-sectional	11649	62.9%	NR	NR	Low-risk
L.Fekadu.A/2022[50]	National	Community	Cross-sectional	9825	60.8%	NR	NR	Low-risk
Melese Getu/2021 [51]	Amhara, SNNP&Oromia	Community	Cross-sectional	1199	69.9%	14.8	NR	Low-risk
Bezie&Addisu/2019 [52]	Amhara	Community	Cross-sectional	373	44.8%	17	17±3.2	Low-risk
Mohammed Abdumalik/2018 [53]	Oromia	Community	Cross-sectional	385	48.8%	NR	NR	Low-risk
Setognal Birara/2021 [54]	Amhara	Community	Cross-sectional	2887	73%	NR	NR	Low-risk
Tezera Abebe/2019 [55]	National	Community	Cross-sectional	9262	64.2%	NR	NR	Low-risk
Tessema /2020 [56]	National	Community	Cross-sectional	11464	62.8%	17.1	17.1±2.5	Low-risk

### Meta-analysis

#### Prevalence of early marriage

**Fig 2:**
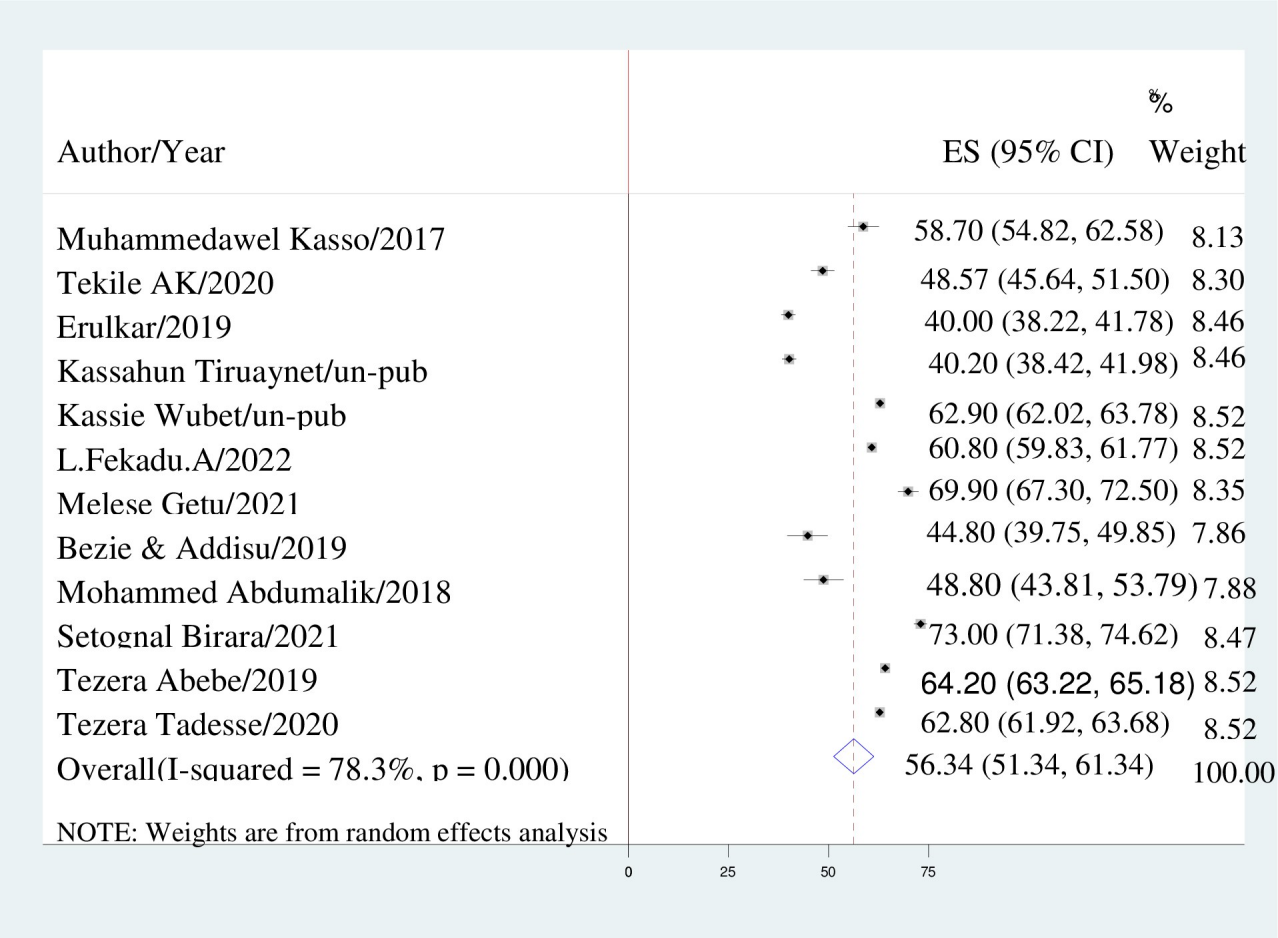
Forest plot displaying the pooled prevalence of early marriage of women in Ethiopia.

The present study employed a random-effects model to estimate the prevalence of early marriage among women in Ethiopia. The overall estimate of early marriage among Ethiopian women was found to be 56.34% (95% CI: 51.34, 61.34), as depicted in Fig **2**.

#### Subgroup analysis

**Fig 3:**
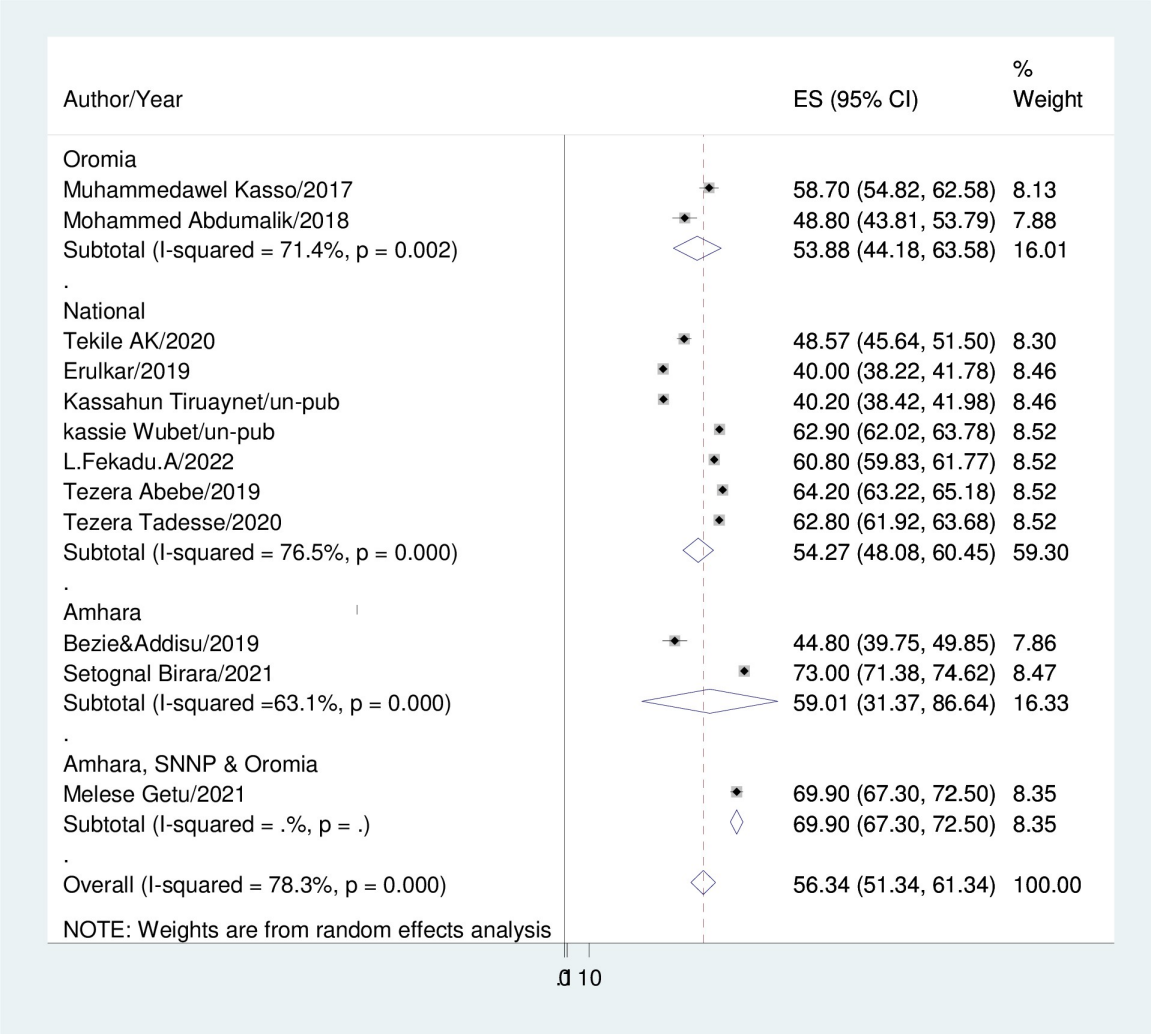
Forest plot displaying the pooled prevalence of early marriage of women in Ethiopia.

Because of the significant heterogeneity that this meta-analysis indicated, subgroup analysis based on sample size and study region was conducted. The Amhara region exhibited the highest prevalence of early marriage, with a rate of 59.01%, whereas the Oromia region demonstrated the lowest incidence, with a prevalence rate of 53.88% (Fig **3**).

**Fig 4:**
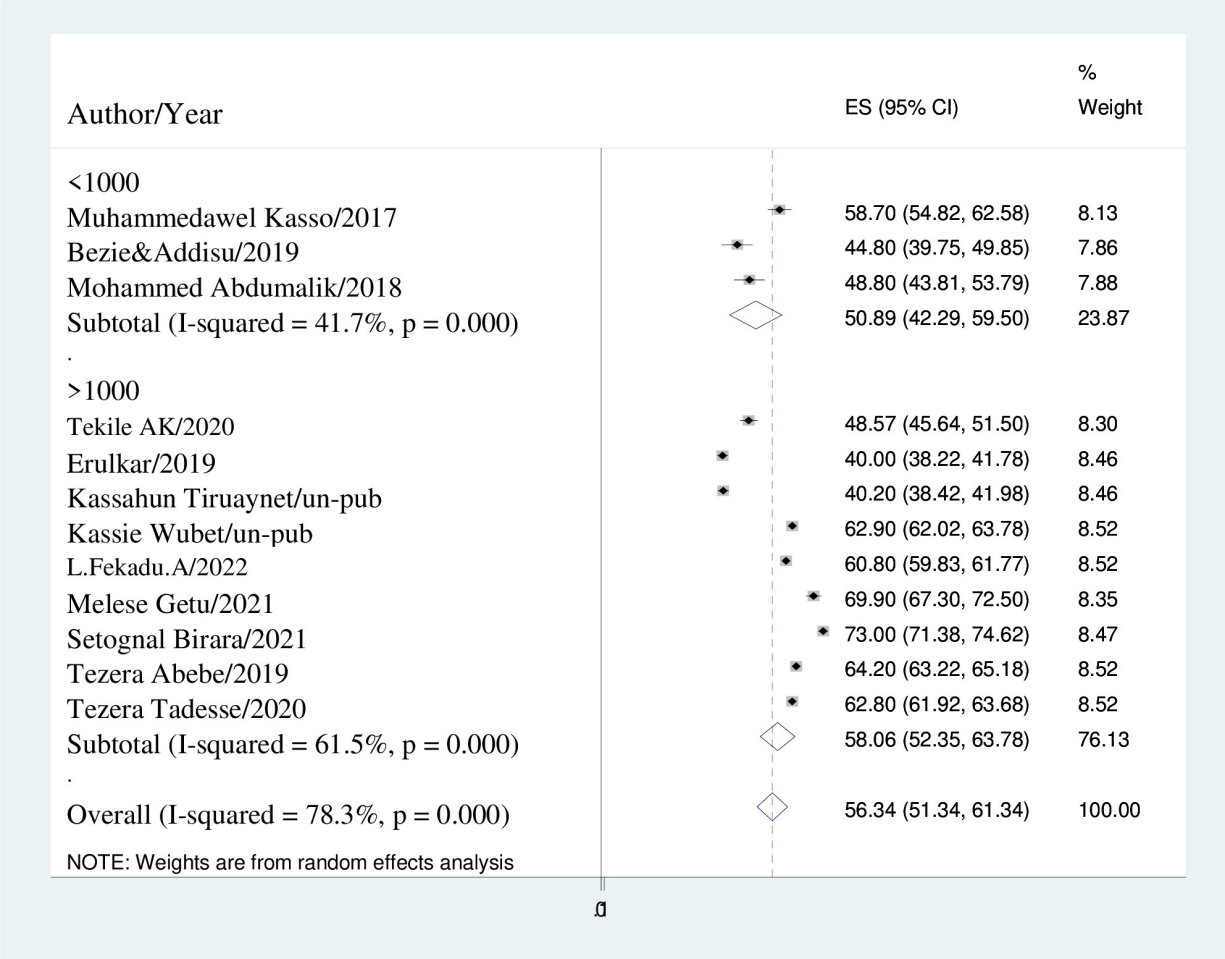
Forest plot displaying subgroup-analysis based on sample size.

Research conducted with sample sizes exceeding 1000 individuals demonstrated a greater incidence of early marriage, with a prevalence of 58.06% (95% CI: 52.35, 63.78); I^2^ = 61.5%. Conversely, studies with sample sizes below 1000 indicated a prevalence of 50.89% (95% CI: 42.29, 59.50); I^2^ = 41.7% (Fig **4**).

#### Heterogeneity and publication bias

A sub-group analysis was conducted to adjust the stated heterogeneity (I^2^ = 78.3%) of the study, based on sample size and study region. Additionally, a univariate meta-regression was performed to identify the primary sources of heterogeneity, with sample size and year as factors. The results of the meta-regression indicated that sample size had a significant impact on the variability observed between studies (P = 0.000) (**Table 2**).

**Table 2:** Meta-regression analysis of factors affecting between-study heterogeneity.

Heterogeneity source	Coefficient’s	Standard error	p-value
Sample size	4.293214	3.068427	0.000
Year	-63.74237	68.4078	0.945

**Fig 5:**
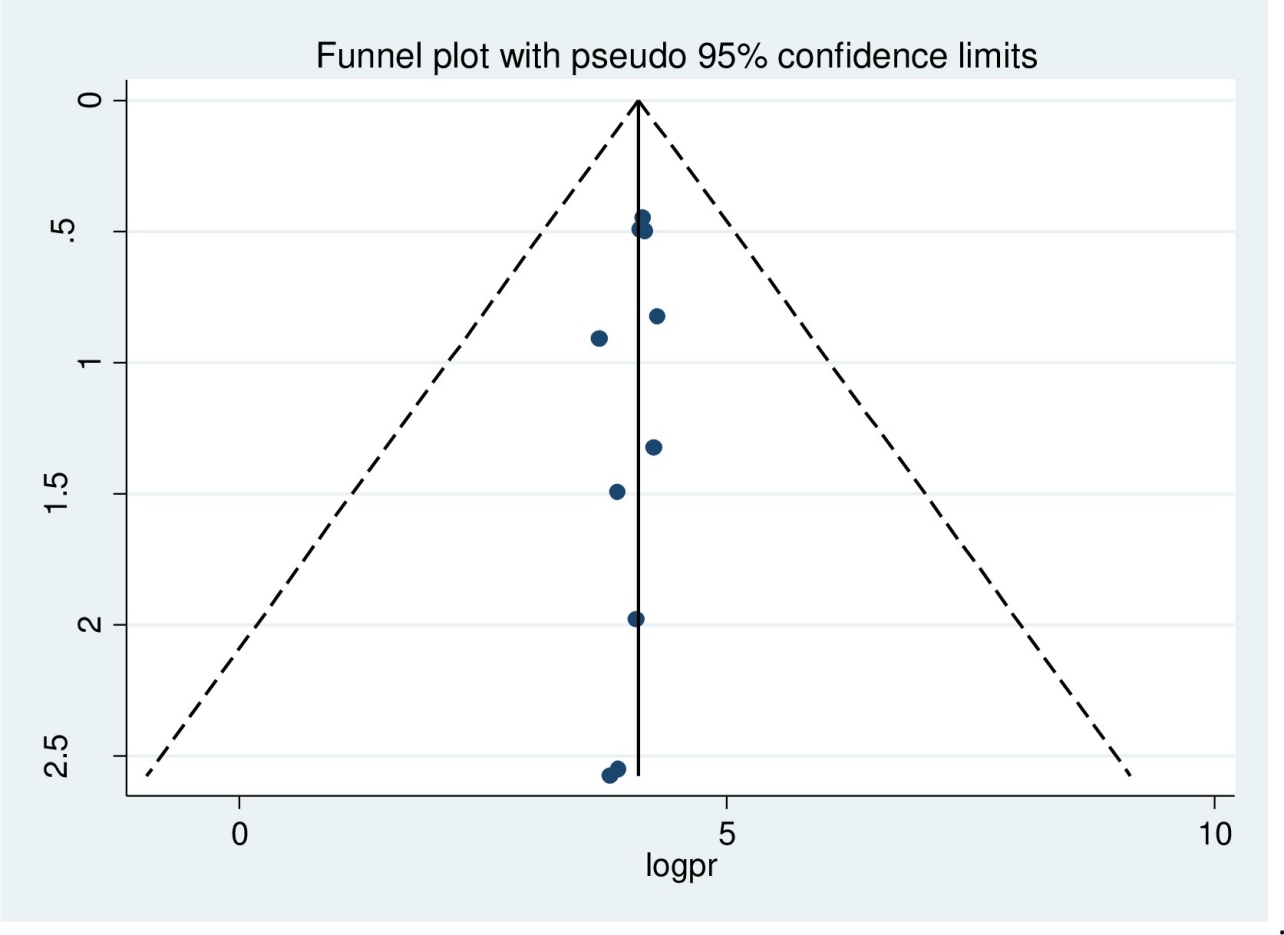
Funnel plot showing symmetrical distribution of studies indicating the absence of publication bias.

The present study demonstrated a visual analysis of publication bias through the use of a funnel plot, while the statistical analysis was conducted using Egger’s test and Begg’s test. The funnel plot revealed a homogeneous distribution of research, as visually observed in Fig **5**.

The findings of the Egger test (p = 0.184) and the Begg test (p = 0.193) did not provide any indication of the presence of publication bias.

#### Leave–one-out-sensitivity analysis

A sensitivity analysis utilizing the leave-one-out method was conducted to ascertain the impact of individual studies on the aggregate prevalence of early marriage among women. Each study was excluded in turn, and the results of the analysis indicated that there was no discernible alteration in the overall prevalence of early marriage among women in Ethiopia (**Table 3**).

**Table 3:** The pooled prevalence of early marriage of women in Ethiopia when one study omitted from the analysis a step at a time.

Study omitted	Estimate	95%CI
Muhammedawel Kasso/2017	56.13	50.88–61.39
Alem et.al/2020	56.34	51.34–61.35
Tekile AK/2020	57.05	51.87–62.23
Sileshi Workineh/2015	56.34	51.34–61.35
Erulkar/2019	57.92	53.56–62.27
Kassahun Tiruaynet/un-pub	57.90	53.52–62.28
kassie Wubet/un-pub	55.71	49.80–61.61
L.Fekadu.A/2022	55.90	50.06–61.75
Melese Getu/2021	55.11	49.88–60.33
Bezie&Addisu/2019	57.33	52.16–62.50
Mohammed Abdumalik/2018	56.99	51.79–62.19
Setognal Birara/2021	54.81	49.80–59.83
Tezera Abebe/2019	55.60	49.87–61.31
Tezera Tadesse/2020	55.72	49.82–61.62
Combined	56.34	51.34–61.35

#### Factors associated with early marriage

The present study examined the potential variables that may contribute to early marriage among women, including lack of formal education, rural residency, parental decision-making, and primary and secondary educational attainment. The results indicated a significant association between early marriage and maternal educational status, as well as rural residency and parental decision-making.

#### No formal educational status

**Fig 6:**
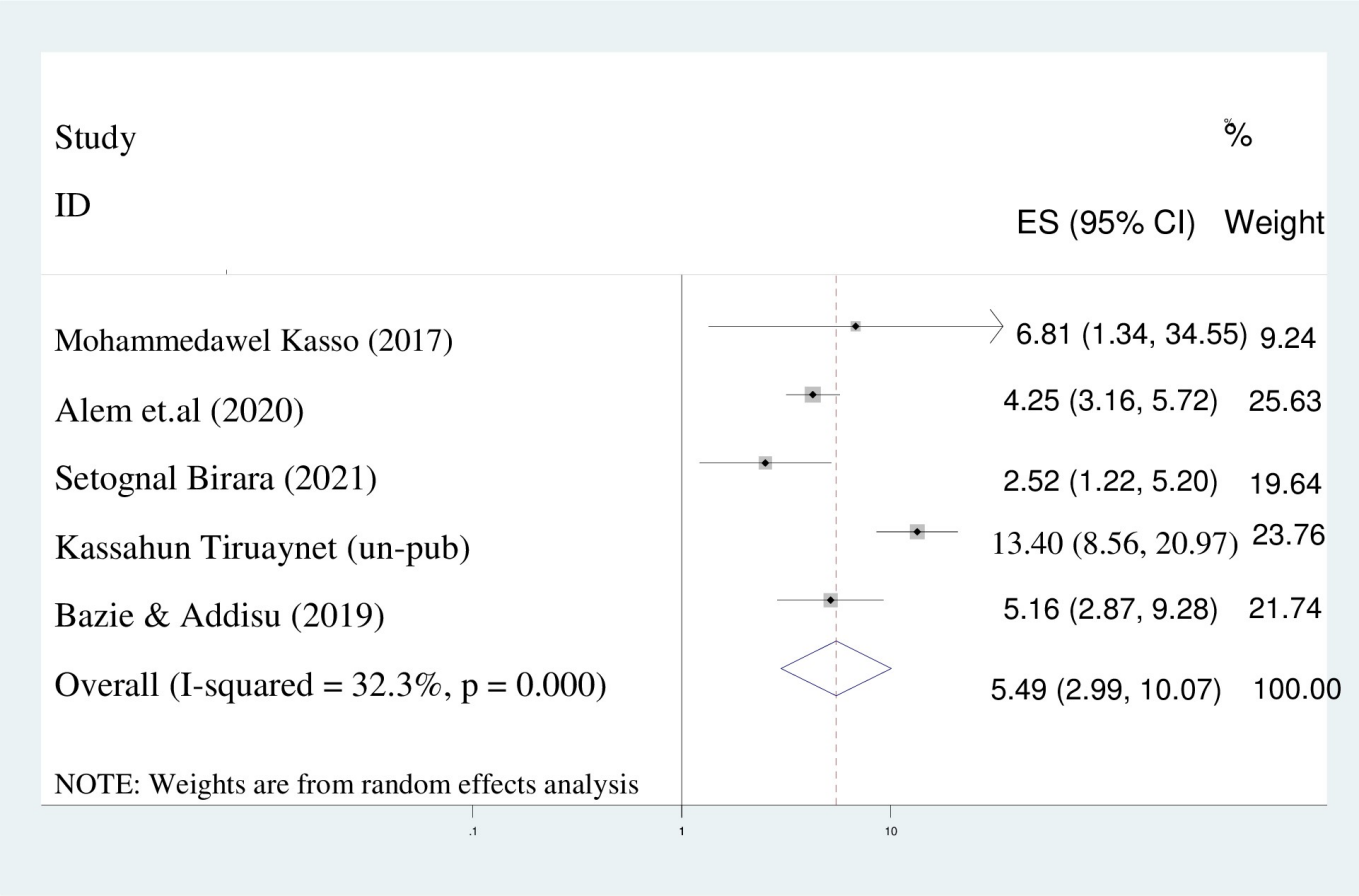
Pooled odds ratio displaying the association of no formal education with early marriage.

Based on the findings of this investigation, it was determined that women lacking formal education were at a significantly higher risk of marrying at an earlier age, with a 5-fold increase in likelihood compared to their formally educated counterparts (AOR = 5.49; 95%CI: 2.99, 10.07). In light of the observed heterogeneity between studies (I^2^ = 32.3%), a random effect model was employed (Fig **6**).

#### Primary educational status

**Fig 7:**
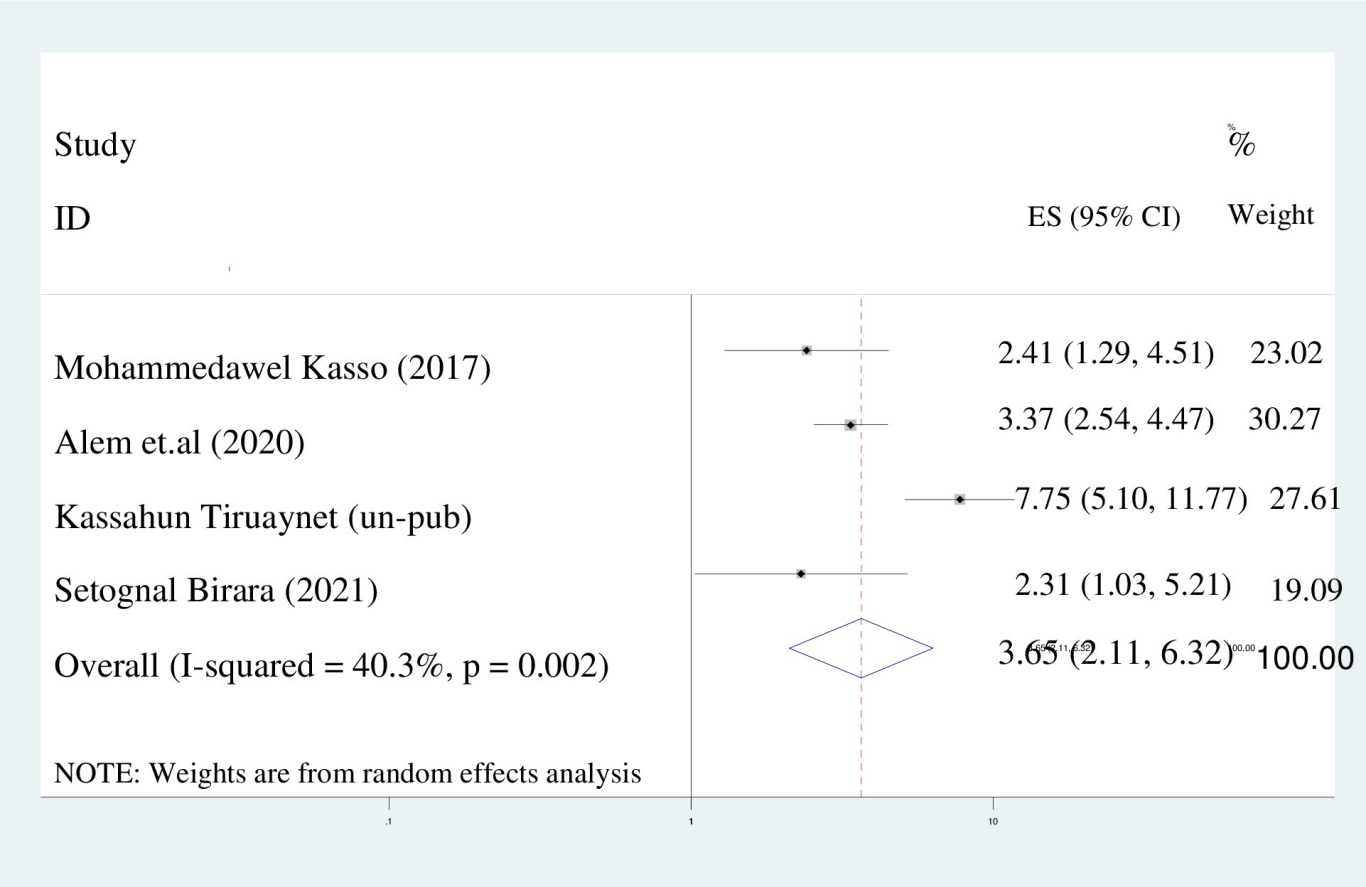
Pooled odds ratio displaying the association of primary education with early marriage.

The present meta-analysis has revealed that women with a primary level of education exhibit a 3.7-fold higher likelihood of early marriage compared to those with secondary and higher educational status (adjusted odds ratio [AOR] = 3.65; 95% [CI]: 2.11, 30.6.32). A random effects model was employed due to the observed I^2^ value of 40.3% (Fig **7**).

#### Secondary educational level

**Fig 8:**
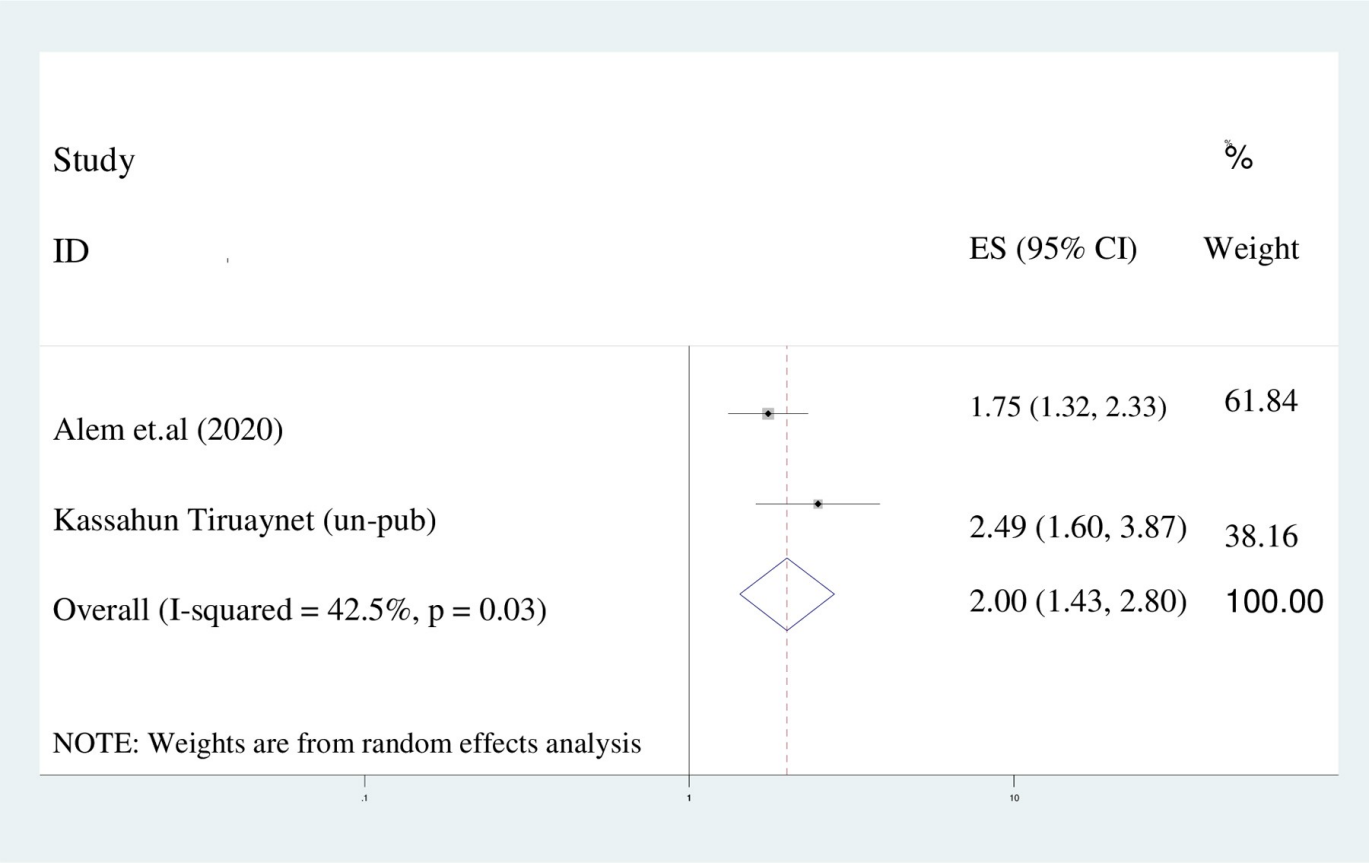
Pooled odds ratio displaying the association of secondary education with early marriage.

The present investigation revealed that individuals with a secondary level of education exhibited a 2.5-fold increase in the likelihood of early marriage compared to their peers (AOR = 2.49; 95%CI: 1.60, 3.87). Given the presence of heterogeneity, as indicated by the I^2^ statistic (42.5%), a random effects model was employed for the analysis (Fig **8**).

#### Rural residency

**Fig 9:**
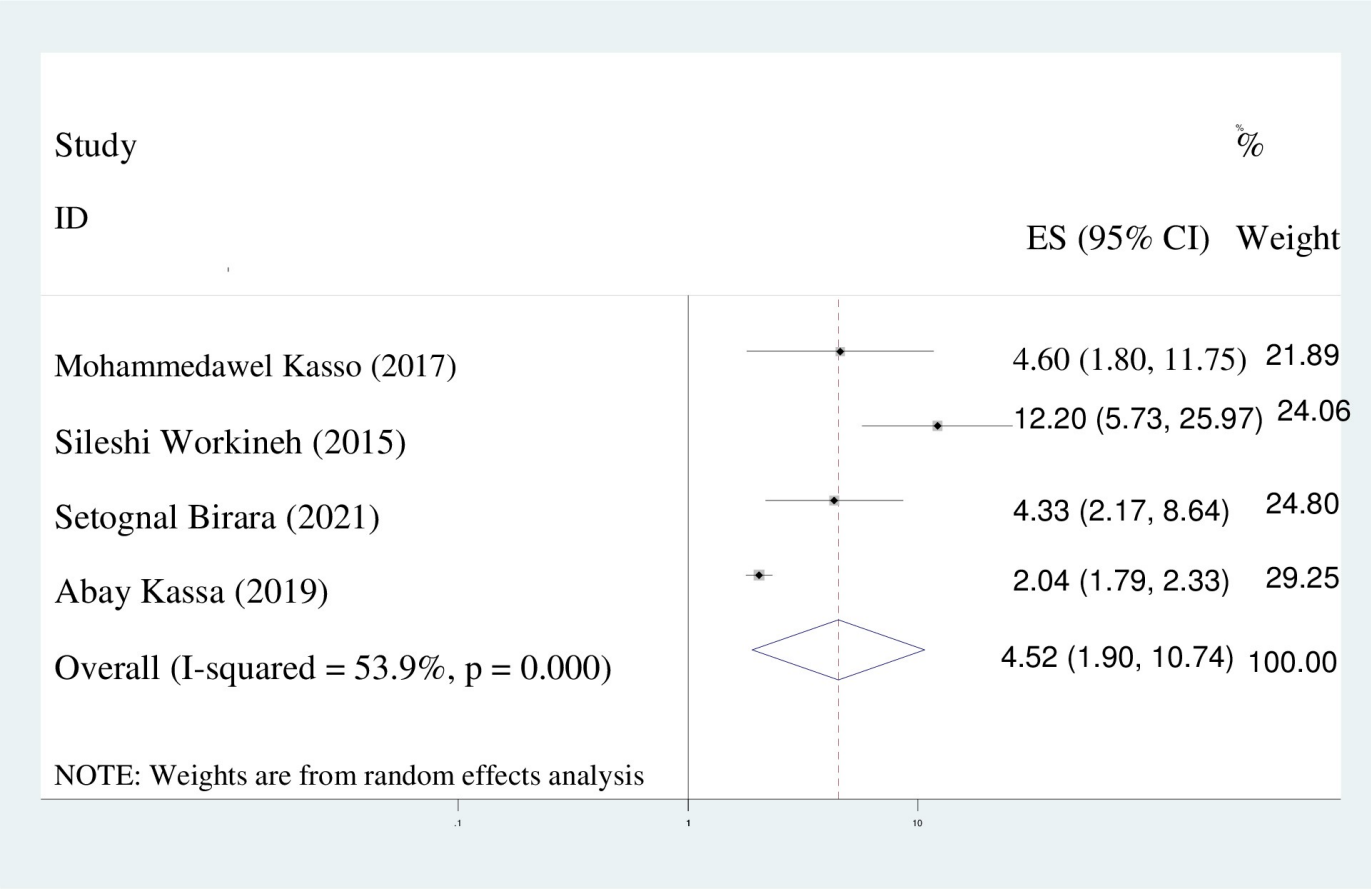
Pooled odds ratio displaying the association of rural residency with early marriage.

The probability of early marriage among women residing in rural areas was found to be 4.5 times greater than that of their urban counterparts (adjusted odds ratio [AOR] = 4.52; 95% [CI]: 1.90, 10.74). In light of the observed heterogeneity (I^2^ = 53.9%), a random effects model was employed **(**Fig 9).

#### Decisions made by parents

**Fig 10:**
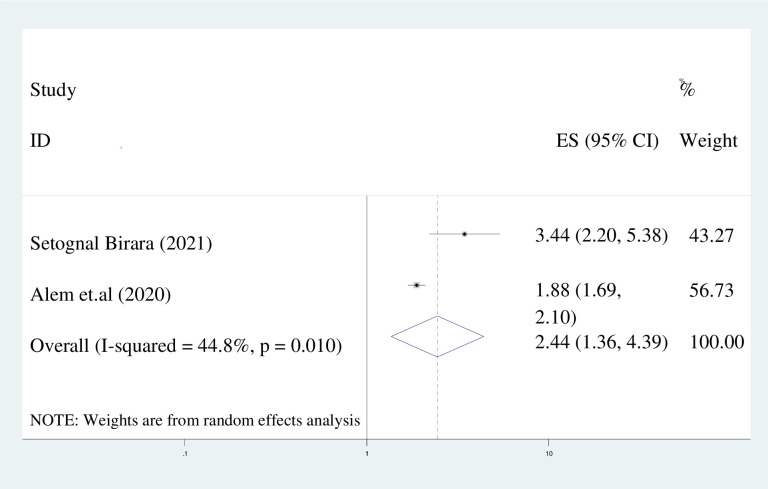
Pooled odds ratio displaying the association of decision made by parents with early marriage.

The present study found that women whose marriage decisions were made by their parents were twice as likely to experience early marriage compared to those who made their own decisions (adjusted odds ratio [AOR] = 2.44; 95% [CI]: 1.36, 4.39). A random effects model was employed due to the observed heterogeneity (I^2^ = 44.8%) **(**Fig **10).**

## Discussion

The ramifications of early marriage are not limited to the young girls themselves, but also extend to subsequent generations, rendering them more vulnerable to morbidity and mortality. Ethiopia, particularly in its rural regions, exhibits alarmingly high rates of child marriage [59], a practice that is deemed unethical and can have deleterious effects on one’s physical, mental, emotional, and intellectual well-being. Both male and female individuals who partake in this practice are deprived of educational and personal growth opportunities [3]. The present systematic review and meta-analysis aimed to ascertain the overall prevalence of early marriage among Ethiopian women and identify its determinants. The key findings were that the national pooled estimate of early marriage and early marriage were substantially correlated with maternal educational status, living residency, and the role of parents’ involvement in marriage decision-making. As a result, 56.34% (95% CI: 51.34–61.34) of Ethiopians were found to have married before the age of 18. This result is in line with research from Mali (58.2%) [60] and Sub-Saharan Africa (55.11%) [61]. This may be due to the similar socioeconomic characteristics of the research locations.

The finding of the present study is lower than studies done in Bangladesh (78.9%) [62]. On the contrary, the result of this study is higher than those from Sudan (45.9%) [37], Iran (18.2%) [63], Egypt (36.7%) [64], Palestinian (41.4%) [65], Nigeria (41.5%] [66], and Nepal (49.6%] [67]. The most likely explanations include the fact that the current study is the most recent, the fact that the majority of the preceding studies were conducted on married women over the age of 20, variations in the legal age of first marriage, and sociocultural variations between countries. Additionally, our study is made up of multiple nationwide surveys that included the majority of rural areas with a high population where child marriage was probably common. This effectively conveys the significance of spreading reproductive health messages to girls.

The present investigation has also shown that the prevalence of early marriage varied based on sample size and administrative region. Consequently, the subgroup analysis conducted based on region demonstrated that the Amhara region exhibited the highest prevalence of early marriage, with a rate of 59.01%. The possible rationale for this finding could be attributed to the fact that within the region, early marriage is deeply ingrained in religious and cultural norms, where premarital sexual activity is perceived as detrimental to a girl’s eligibility for marriage. This is due to the belief that a girl’s worth is contingent upon her sexual purity, her future role as a devoted wife and mother, and her commitment to upholding family honor [68].

In this meta-analysis, early marriage was predicted by lack of formal education, elementary and secondary education levels, rural location, and parental marriage decision. Young girls who did not have formal schooling were 5.5 times more likely to get married young than those who did. The earlier research confirmed this finding. Studies found that women’s education level was an independent predictor of early marriage in Malawi [69]. This may be because education gives people the knowledge they need to understand their rights and decide on marriage with knowledge [70]. In contrast, those who had no formal education can’t exercise their legal age of marriage which intern leads to early marriage.

The study found that compared to women with higher education, women with primary and secondary education were 3.6 and 2.5 times more likely to be married before the age of 18 respectively. Our findings are consistent with those from Sudan [37], Iran [63], and Egypt [64] which found that better educational level is strongly related to early marriage, in contrast to research conducted in Mali [60] that found the opposite. The cause may be because less educated women lack awareness about the ideal marriage age and the negative effects of early marriage on one’s health.

In this study, rural women had a 4.5 times higher probability of getting married young than urban women did. This conclusion is consistent with research done in Serbia [36] and Bangladesh [71]. This may be explained by the fact that women in rural areas may not be aware of the negative effects of early marriage on their health, education, and economic status [72]. Additionally, when their parents or guardians violate their human rights as stated by national family law, they are unsure of where to turn [73]. As a result, women who lived in rural areas of the region were more likely to get married young than women who lived in the region’s urban areas.

Compared to women whose first marriage decision was made by them, those whose first marriage was decided by their parents or other relatives had a higher likelihood of being married young. Parents may frequently believe that a young girl is a financial burden, which may account for this. They, therefore, believe that marrying their young daughters will aid the poor family in terms of both social and financial rewards [74].

## Conclusions

To sum up, the prevalence of early marriage among Ethiopian women was **high**. The prevalence of early marriage also varied depending on the region and sample size. The Oromia region had the lowest incidence of early marriage, with a prevalence rate of 53.88%, while the Amhara region had the highest prevalence of early marriage at 59.01%. Hence, the enhancement of maternal education and the provision of greater decision-making authority to women, particularly in rural regions, ought to constitute the primary objectives of early marriage policy in Ethiopia, to mitigate and ultimately eradicate the practice of early marriage.

### Strength and limitations

The present study exhibits several strengths. Firstly, it utilized international compressive electronic search engines. Secondly, to mitigate selection bias, a systematic search of the literature was conducted, and research that met well-defined criteria was included. Additionally, preprint articles, which have not yet undergone peer review, were also incorporated. Finally, the study protocol was registered in the international prospective register of systematic reviews. However, the study is not without limitations, as it only included papers published in the English language. This study presents a systematic review and meta-analysis of data about Ethiopia, intending to provide a national estimation. However, it is important to note that the lack of available data from certain regions, namely Benishangul Gumuz, Addis Ababa, Afar, Gambella, Somalia, Dire Dawa, and Harare, may limit the national representativeness of the findings.

## Supporting information

S1 File Prisma checklist. (DOCX)

S2 File Methodological quality assessment of included studies using Joanna Brigg’s Institute quality appraisal criteria scale (JBI). The eight-item questions assessing inclusion criteria, study setting and participant, exposure measurement, objectives, confounder, statically analysis, outcome measurement, and dealing confounder were used.
(DOCX)

S3 File Risk of bias assessment for the included studies. The ten-item questions of which four items assess external and six items assess internal validity were used.
(DOCX)

S1 Dataset (XLSX)
