# Supplementary material for: Early marriage and its associated factors among women in Ethiopia: Systematic reviews and meta-analysis
Source: PLoS One. 2023 Nov 22;18(11):e0292625. doi: 10.1371/journal.pone.0292625 (PMC10664944; doi:10.1371/journal.pone.0292625)
Supplement: S2 File — The eight-item questions assessing inclusion criteria, study setting and participant, exposure measurement, objectives, confounder, statically analysis, outcome measurement, and dealing confounder were used. (DOCX) [file pone.0292625.s002.docx]

Supplementary file2. Quality assessment for the included Studies

| Item | Clearly defined inclusion | Describe study setting and participant | Valid and reliable exposure measurement | Objective and standard criteria for measurement | Identified confounder | Strategies to deal with confounders | Valid and reliable outcome measurement | Appropriate statically analysis | No of ‘yes’s ‘ |
| --- | --- | --- | --- | --- | --- | --- | --- | --- | --- |
| Muhammedawel Kasso | Yes | Yes | No | Yes | Yes | No | Yes | Yes | 6/8=75 |
| Alem et.al | Yes | Yes | Yes | Yes | No | No | Yes | Yes | 6/8=75 |
| Tekile AK | Yes | Yes | No | Yes | Yes | No | Yes | Yes | 6/8=75 |
| Sileshi Workineh | Yes | Yes | No | Yes | Yes | Yes | Yes | Yes | 7/8=87.5 |
| Erulkar | Yes | Yes | No | Yes | Yes | Yes | Yes | Yes | 7/8=87.5 |
| Kassahun Tiruaynet | Yes | Yes | Yes | Yes | Yes | No | Yes | Yes | 7/8=87.5 |
| kassie Wubet | Yes | Yes | No | Yes | Yes | Yes | Yes | Yes | 7/8=87.5 |
| L.Fekadu.A | Yes | Yes | Yes | Yes | No | No | Yes | Yes | 6/8=75 |
| Melese Getu | Yes | Yes | No | Yes | Yes | No | Yes | Yes | 6/8=75 |
| Bezie & Addisu | Yes | Yes | Yes | Yes | Yes | No | Yes | Yes | 7/8=87.5 |
| Mohammed Abdumalik | Yes | Yes | Yes | Yes | No | No | Yes | Yes | 6/8=75 |
| Setognal Birara | Yes | Yes | No | Yes | Yes | Yes | Yes | Yes | 7/8=87.5 |
| Tezera Tadesse | Yes | Yes | Yes | Yes | No | No | Yes | Yes | 6/8=75 |
| Tezera Abebe | Yes | Yes | Yes | Yes | Yes | No | Yes | Yes | 7/8=87.5 |
